# Supplementary material for: How Should We Assign Large Infiltrative Hepatocellular Carcinomas for Staging?
Source: Cancers (Basel). 2020 Sep 10;12(9):2589. doi: 10.3390/cancers12092589 (PMC7564178; doi:10.3390/cancers12092589)
Supplement: Supplementary file 1 [file cancers-12-02589-s001.pdf]

cancers

How Should We Assign Large Infiltrative Hepatocellular Carcinomas for Staging?

Yoo Jin Lee, Yoo Ra Lee, Chung Gyo Seo, Hyun Gil Goh, Tae Hyung Kim, Sun Young Yim, Na Yeon Han, Jae Min Lee, Hyuk Soon Choi, Eun Sun Kim, Bora Keum, Hyonggin An, Beomjin Park, Yeon Seok Seo, Hyung Joon Yim, Ji Hoon Kim, Young Dong Yu, Dong Sik Kim, Yoon Tae Jeen, Hoon Jai Chun, Hong Sik Lee, Chang Duck Kim and Soon Ho Um

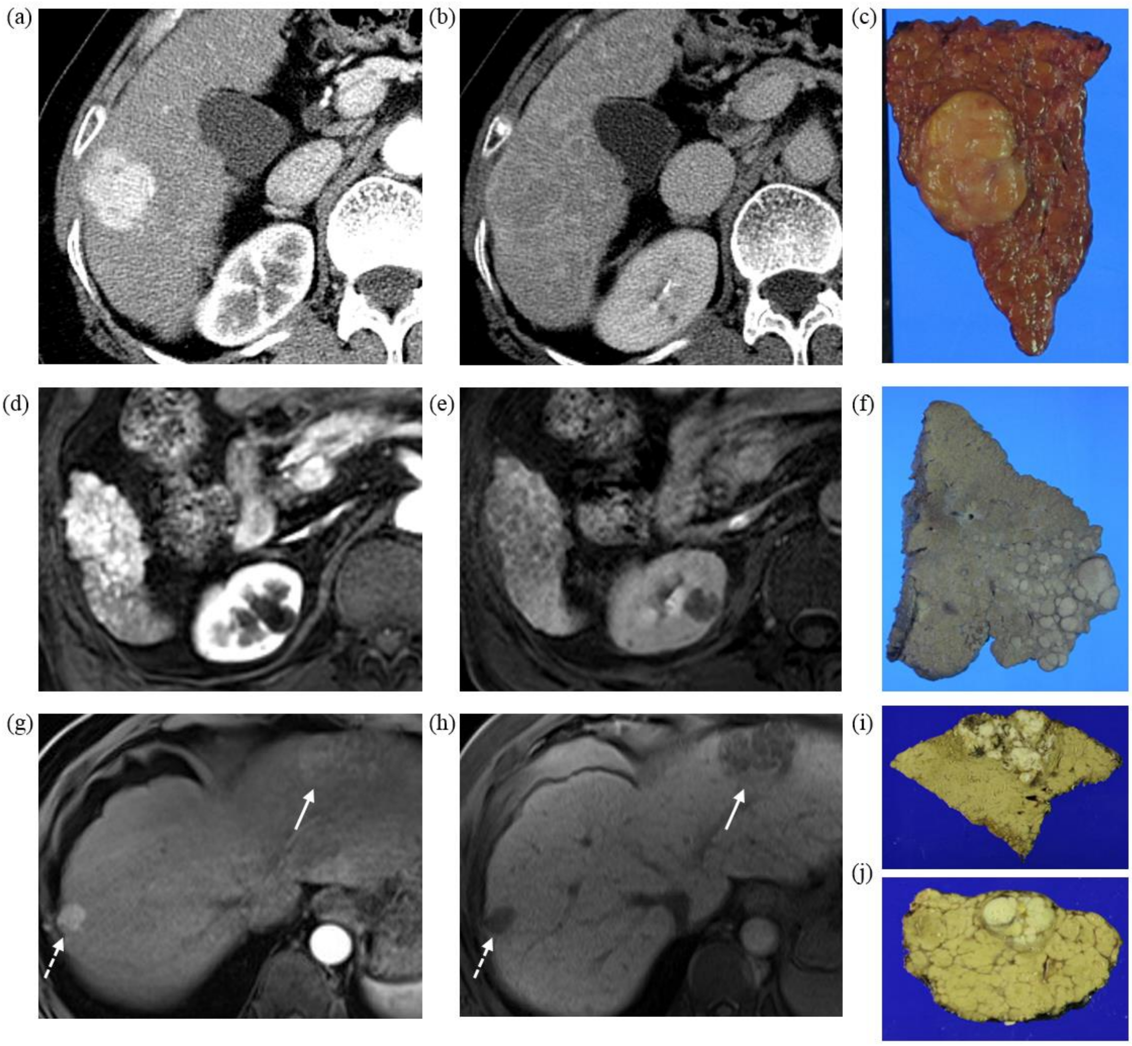

Figure S1. Imaging and gross morphologic subtypes of HCCs. Imaging findings of nodular HCC has typical characteristics of HCC with (**a**) early arterial hyperenhancement, (**b**) washout pattern on portal phase in three phase CT and (**c**) sharp demarcating margin on pathologic finding. Infiltrative HCC staged BCLC A shows (**d**) tumor with miliary pattern of enhancement in early arterial phase T1-weighted MR image, (**e**) wash-out pattern at hepatobiliary phase and (**f**) multiple foci of varying size that fuse to form larger foci without definite margin in pathologic findings. BCLC B staged HCC comprising two HCCs shows (**g**) inhomogeneous enhancing mass in early arterial phase MR image for larger HCC (solid arrow) and enhancing nodule with discrete margin for another smaller HCC (dotted arrow) and (**h**) wash-out pattern at hepatobiliary phase for both HCCs. The pathologic findings corresponding to (**i**) larger HCC shows mass with permeative appearance without distinct margin while (**j**) smaller HCC has discrete margin.

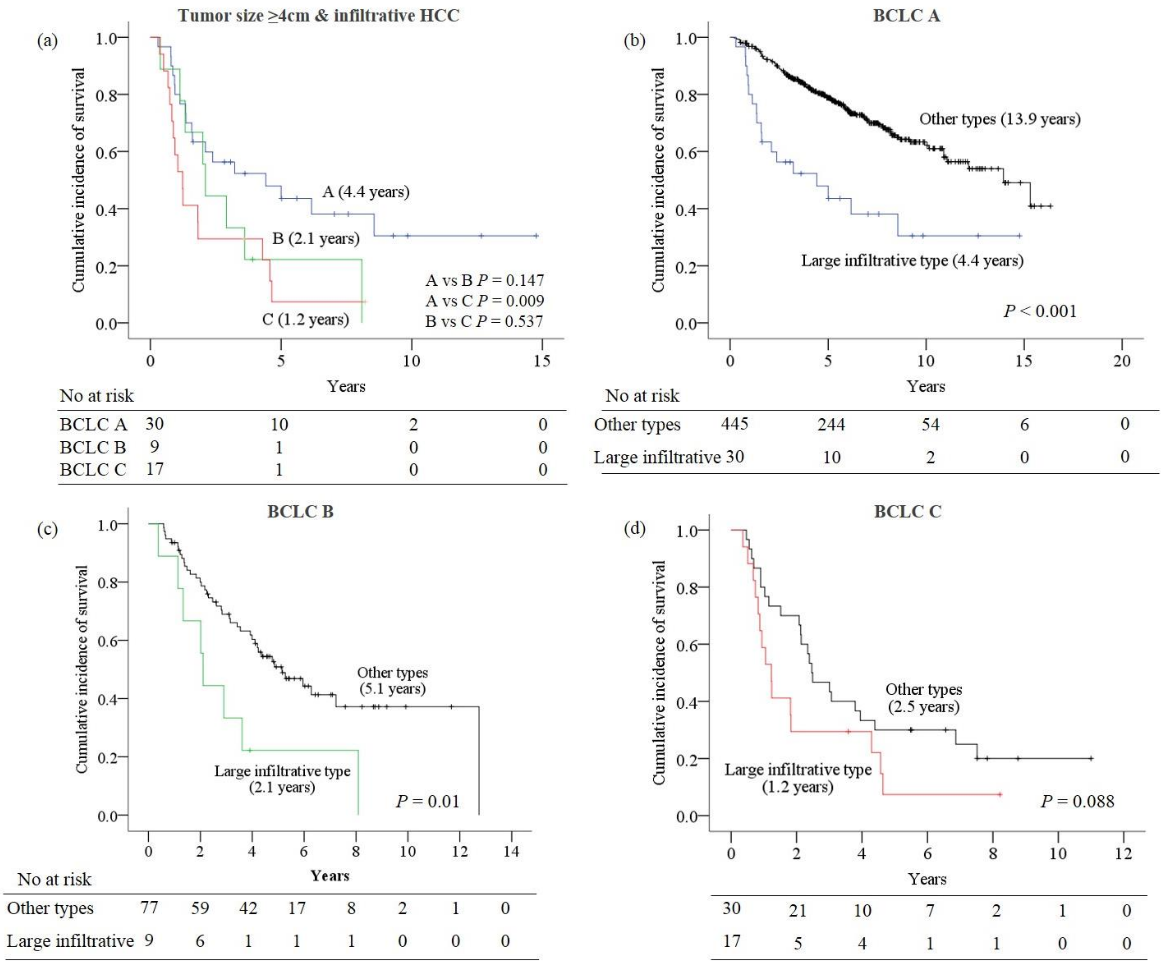

Figure S2. (**a**) Overall survival according to the current BCLC staging system for large infiltrative HCC. Impact of large infiltrative HCC on different stages: (**b**) BCLC A, (**c**) BCLC B, and (**d**) BCLC C. Median survival time is shown in brackets.

© 2020 by the authors. Licensee MDPI, Basel, Switzerland. This article is an open access article distributed under the terms and conditions of the Creative Commons Attribution (CC BY) license (http://creativecommons.org/licenses/by/4.0/).
